# The Effect of Chromium Nanoparticles and Chromium Picolinate in the Diet of Chickens on Levels of Selected Hormones and Tissue Antioxidant Status

**DOI:** 10.3390/ani10010045

**Published:** 2019-12-24

**Authors:** Anna Stępniowska, Aleksandra Drażbo, Krzysztof Kozłowski, Katarzyna Ognik, Jan Jankowski

**Affiliations:** 1 Department of Biochemistry and Toxicology, Faculty of Animal Sciences and Bioeconomy, University of Life Sciences in Lublin, Akademicka 13, 20-950 Lublin, Poland; kasiaognik@poczta.fm; 2 Department of Poultry Science, Faculty of Animal Bioengineering, University of Warmia and Mazury, Oczapowskiego 5, 10-719 Olsztyn, Poland; aleksandra.drazbo@uwm.edu.pl (A.D.); kristof@uwm.edu.pl (K.K.); janj@uwm.edu.pl (J.J.)

**Keywords:** chromium, nanoparticles, hormones, chicken, antioxidant, supplementation

## Abstract

**Simple Summary:**

We have postulated that supplementation with Cr can increase serotonin levels and improve the antioxidant status of chickens, with no adverse effect on the secretion of other hormones. Obtaining such results may be beneficial in high-density rearing of poultry exposed to many stressors. The studies compared the effects of two forms of Cr (Cr-Pic and Cr-NP) found at two doses 3 and 6 mg/kg. It was found that at the dose of 3 mg/kg, the expected beneficial results can be obtained (increase in serotonin and decrease in norepinephrine level), however, due to the deterioration of the antioxidative system, these studies should be continued to verify these results. Due to the adverse effect of Cr at 3 mg/kg on the antioxidant status of chickens, this level of Cr should not be considered in both forms Cr-Pic and Cr-NP as a feed additive for broiler chickens. In the future, studies on the potential beneficial effects of Cr on the organism should take into account doses lower than 3 mg/kg.

**Abstract:**

We have postulated that supplementation with Cr can increase serotonin levels and improve the antioxidant status of chickens, with no adverse effect on the secretion of other hormones. The study aimed to determine what form and dose of Cr more favorably affect the level of selected hormones (insulin, glucagon, serotonin, dopamine, noradrenaline, histamine, T3 and T4) and the antioxidant status (level of malondialdehyde and lipid peroxides, activity of superoxide dismutase and catalase) of chicken tissues. The experiment was carried out on chickens randomly divided into five treatment groups. The basal diets (control group) were supplemented with two levels of Cr (3 and 6 mg/kg) and two Cr sources: Cr-picolinate (Cr-Pic) and Cr-nano (Cr-NP) to obtain four experimental diets: 3 mg/kg Cr-Pic, 6.0 mg/kg Cr-Pic, 3.0 mg/kg Cr-NP. and 6.0 mg/kg Cr-NP. The addition of Cr in both forms increased the level of serotonin at a dose of 3 mg/kg and, at the same time, reduced the level of noradrenaline. The addition of Cr at 3 mg/kg, irrespective of the form used, regulated the level of hormones of carbohydrate metabolism (increasing insulin levels and reducing glucagon levels) and had an adverse effect on the antioxidant status of the liver and breast muscle. Due to the adverse effect of Cr at 3 mg/kg on the antioxidant status of chickens, this level of Cr should not be considered in both forms Cr-Pic and Cr-NP as a feed additive for broiler chickens. In the future, studies on the potential beneficial effects of Cr on the organism should take into account doses lower than 3 mg/kg.

## 1. Introduction

Studies conducted on laboratory animals [1,2] and a few on farm animals [3,4] show that chromium (Cr) may affect the secretion of certain hormones, mainly insulin, glucagon, and serotonin. Chromium in the form of picolinate (Cr-Pic) has been shown to reduce blood glucose in laboratory animals and in diabetes patients by increasing insulin secretion [5,6]; alleviate symptoms of depression [7], and reduce cortisol levels in laboratory animals [8]. Research in rats [8] has shown that Cr increases the peripheral availability of tryptophan, an amino acid essential for serotonin synthesis in the brain (5-HT). Sahin et al. [9] reported reduced corticosterone levels in broiler chickens kept under heat stress conditions and, at the same time, receiving a Cr-Pic supplement. Both increased serotonin levels and reduced corticosterone levels suggest that Cr may have a stress-relieving effect. According to Sahin et al. [9], chromium may also increase secretion of thyroid hormones triiodothyronine (T3) and thyroxine (T4) in heat-stressed chickens. Thyroid hormones are considered to be growth stimulants in poultry [9,10,11,12]. According to Sahin et al. [9], the addition of Cr to the diet of chickens increases the levels of hormones T3 and T4 and, at the same time, improves the growth performance of broiler chickens kept under stress. There have been many studies [9,13] suggesting that Cr can be used during heat stress, but they do not explain the mechanism whereby Cr alleviates stress responses and improves the growth of chickens under stress. In addition, there is some concern that the wrong forms and doses of Cr may interfere with the functions of other hormones, such as histamine, insulin, or glucagon. Chromium (III) is a component of glucose tolerance factor (GTF), which takes part in glucose metabolism by enhancing the effect of insulin. Cr improves the effectiveness of insulin by increasing its binding to receptors and thereby increasing the sensitivity of target cells [14,15].

Cr is believed to be necessary for the activation of certain enzymes and for the stabilization of proteins and nucleic acids, and for this reason, it is considered to display antioxidant activity [16]. A study by Tezuka et al. [17] found that Cr protects rats against oxidative damage associated with carbon tetrachloride, while Preuss et al. [18] noted reduced lipid peroxidation in isolated hepatocytes of rats receiving supplementation with chromium picolinate and nicotinate. In hypertensive rats receiving Cr as picolinate, TBARS in the liver and kidneys decreased as well [18]. The mechanism of the antioxidant effect of Cr is still not fully understood and requires further research. 

Increasingly, the sale of poultry feed additives includes preparations containing various forms and doses of Cr. Literature data suggest that Cr can have both positive and negative effects, and no safe dose and form have been established so far. Cr-based feed additives currently available for sale are recommended as stress-relieving supplements, e.g., heat or transport stress. We believe that our research will deepen our knowledge and allow us to determine whether preparations with Cr can be used in the range of 3–6 mg/kg as follows from the literature data or, for example, you cannot exceed the level of 3 mg/kg as shown in our previous studies [19]. A detailed assessment of the impact of Cr on hormonal balance and tissue redox status has not been presented in the literature so far and can certainly help determine whether Cr is safe for the organism and whether it should be used in poultry at all. We have postulated that supplementation with Cr can increase serotonin levels and improve the antioxidant status of chickens, with no adverse effect on the secretion of other hormones, including neurotransmitters. The study aimed to determine what form (Cr-Pic or Cr-nano (Cr-NP)) and dose of Cr (3 or 6 mg/kg) more favorably affect the level of selected hormones (insulin, glucagon, serotonin, dopamine, noradrenaline, histamine, T3 and T4) and the antioxidant status of chicken tissues.

## 2. Material and Methods

### 2.1. Nanomaterial 

Chromium nanopowder (Cr-NP) with purity 99.9%, size 60~80 nm, spherical shape, specific surface area 6–8 m^2^/g, bulk density 0.15 g/cm^3^, and true density 8.9 g/cm^3^ was purchased from SkySpring Nanomaterials (Houston, TX, USA). 

### 2.2. Animals and Diets

The experiment was carried out in a poultry house at the experimental facilities of the Department of Poultry Science, University of Warmia and Mazury in Olsztyn, Poland. Day-old male Ross 308 chickens (405 male birds) were randomly divided into 5 treatment groups, with nine replicates of 9 birds each. The control group received a basal diet without any additives. Group 3 mg/kg Cr-Pic received 3.0 mg/kg of Cr-picolinate, group 6 mg/kg Cr-Pic—6.0 mg/kg of Cr-picolinate, while group 3 mg/kg Cr-NP—3.0 mg/kg of Cr-nano and group 6 mg/kg Cr-NP—6.0 mg/kg of Cr-nano. The experimental additives were added to the feed mixtures in the form of suspensions in rapeseed oil (0.5%). The nutritional value of all experimental diets corresponded to the nutrient requirements of broiler chickens (Aviagen, 2014). Birds were kept in cages, and each cage was equipped with nipple drinkers and a feeder that was manually filled daily. All chickens had free access to feed and water. The heating and light program was in accordance with the Ross Broiler Management Manual (Aviagen, 2014). The experimental procedure was approved by the Local Animal Experimentation Ethics Committee in Olsztyn (Mo. 30/2015).

The chemical composition of the basal starter (0–21 days) and grower/finisher (22–35 days) diets is shown in Table 1.

### 2.3. Growth Trial and Sample Collection

During the experiment, body weight gain (BWG) and feed consumption were recorded and calculated on a pen basis. Daily feed intake (ADFI) per bird was calculated based on total feed consumption for the pen for the entire experimental period divided by the number of days in the period. The feed conversion ratio (FCR; kg of feed/kg of body weight gain) was calculated on a pen basis from body weight gain and feed consumption [19].

At 35 days of age, 9 birds representing the average body weight of each group were selected, tagged, and fasted for 8 h. Blood samples were taken from 9 birds from each group (one bird for each replication). Then 9 broilers per group (one bird representing the average body weight per pen) were killed at a slaughterhouse. The birds (without being transported) were electrically stunned (400 mA, 350 Hz), hung on a shackle line, and exsanguinated by a unilateral neck cut severing the right carotid artery and jugular vein. After a 3-min bleeding period, the birds were scalded at 61°C for 60 s, defeathered in a rotary drum picker for 25 s, and manually eviscerated. The breast muscles and liver were collected for further analysis.

### 2.4. Laboratory Analysis

Mineral (Ca, Mg, Fe, Cu, and Zn) content in the blood samples and Cr content in feed, liver, and breast muscle samples was determined by flame atomic absorption spectrometry (F AAS). Kits produced by Cell Biolabs, Inc. (San Diego, CA, USA) were used to determine the level of the following hormones: insulin, glucagon, serotonin, dopamine, noradrenaline, histamine, T3 and T4. 

In the liver and breast muscle of the chickens, the activity of superoxide dismutase (SOD) and catalase (CAT), and the concentration of lipid peroxides (LOOH) and malondialdehyde (MDA) were determined. The adrenaline assay by Greenwald [20], for determination of superoxide dismutase, was modified at 320 nm to increase the selectivity of transient reaction products at this wavelength [21], whereas catalase was assayed according to the method of Bartosz [21]. Lipid peroxides were determined according to the method of Buege and Aust [22] and malondialdehyde according to the method of Salih et al. [23]. 

### 2.5. Statistical Analysis

For performance parameters, each replicate pen (*n* = 9) was considered as an experimental unit for the statistical analysis. For analysis of other parameters, individual birds (*n* = 9) were considered as experimental units. The Statistica software package version 13.1 (Statsoft Inc., Kraków, Poland, 2016) was used to determine whether variables differed between treatment groups. The comparison of the control group vs. all other groups was performed by planned contrast analysis. Two-way ANOVA was performed to assess the effects of the supplementation levels of chromium, the source of chromium, and the interaction between the level and source of chromium (dose x source). When the ANOVA indicated significant treatment effects, means were separated using Tukey’s multiple range test. The results are presented in the tables as mean values with pooled standard errors. Data were checked for normal distribution before the statistical analysis was performed. Differences were considered to be significant at *p* ≤ 0.05.

## 3. Results

In the whole rearing period, the control group consumed 2.246 mg Cr/kg BW. In the liver of birds, 0.454 µg Cr/g was noted, whereas in the breast muscle, 0.168 µg Cr/g. In the groups receiving Cr at 3 mg/kg, hepatic accumulation of Cr was 18.5% more than in the control group and 2.9% more in the breast muscle than in the control group. In groups receiving Cr at a dose of 6 mg/kg in the liver accumulated by 21.4% more Cr, and in the breast muscle by 3.9% more Cr than in the control group. According to the USEPA, the maximum permissible limit of Cr in chicken meat is 1 mg/kg and has not been exceeded [24].

Compared to the control group, the addition of Cr to the chicken diet in the amount of both 3 and 6 mg/kg, irrespective of the form, resulted in a lower final body weight (2.08 vs. 1.99 vs. 2.00 kg, *p* = 0.033), lower ADFI (86.7 vs. 82.5 vs. 82.8 g/bird/day, *P* = 0.046), and lower livability (98.77 vs. 98.77 vs. 97.53 %, *p* = 0.042). The Cr source was found to have no effect on growth performance [19]. 

An increase in insulin content was noted in the group receiving 6 mg/kg Cr-NP relative to the control group (*p* < 0.001). Compared to the control group, the addition of Cr at 3 and 6 mg/kg, irrespective of the form used, increased the content of serotonin (*p* < 0.001), while reducing the content of noradrenaline (*p* = 0.001). Two-way ANOVA showed dose x source interactions for serotonin and insulin levels, because increasing Cr-NP from 3 to 6 mg/kg of feed resulted in an increase in serotonin and insulin while increasing Cr-Pic did not. A decrease in glucagon (*p* = 0.016) and histamine (*p* < 0.001) content was noted in the groups receiving Cr-Pic at 3 and 6 mg/kg and Cr-NP at 3 mg/kg relative to the control group. Compared to the control group, the addition of Cr-NP irrespectively of the dose used, increased the content of dopamine (*p* < 0.001), while the addiction of Cr-Pic at dose 6 mg/kg increased the content of T4 (*p* = 0.029). The addition of Cr in the form of Cr-NP increased the content of dopamine more than in the form of Cr-Pic (*p* < 0.001) (Table 2).

Compared to the control group, the addition of 3 and 6 mg/kg Cr to the diet of chickens, irrespectively of form used, caused an increase in the plasma content of Ca and Fe (*p* < 0.001, both), while reducing the content of Mg (*p* = 0.001) and Zn (*p* < 0.001). A decrease in Cu content (*p* < 0.001) was noted in the group receiving 6 mg/kg Cr-Pic and 3 and 6 mg/kg Cr-NP relative to the control group. Increasing the Cr dose to chickens’ diet from 3 to 6 mg/kg contributed to an increase in Fe content (*p* < 0.001) and a decrease in Cu content (*p* <0.001) in blood plasma. The addition of Cr as Cr-NP was found to increase the plasma content of Fe (*p* < 0.001) and to reduce the content of Cu and Zn (*p* < 0.001, both) more than Cr-Pic (Table 3).

Compared to the control group, the addition of Cr-Pic at 3 mg/kg caused a decrease in LOOH content (*p* = 0.023) in chickens’ livers, while the addition of 6 mg/kg Cr-Pic and 3 and 6 mg/kg Cr-NP caused an increase in LOOH content (*p* = 0.008) in breast muscle. An increase in MDA content was noted in groups receiving 6 mg/kg Cr-Pic (in liver) and 3 and 6 mg/kg Cr-NP (in liver and breast muscle) (*p* = 0.004, *p* = 0.043, respectively) relative to control group. Irrespective of the form of Cr used, increasing the addition of this element from 3 to 6 mg/kg of feed caused an increase in LOOH content (*p* = 0.031) and in MDA content (*p* = 0.012) in the liver. Indicators of lipid peroxidation were higher in the liver and breast muscle of chickens from the Cr-NP treatments, i.e. LOOH (*p* = 0.002 and *p* = 0.006) and MDA (*p* = 0.003 and *p* = 0.033). Compared to the control group, the addition of Cr-Pic at 3 and 6 mg/kg caused a decrease in SOD activity (*p* < 0.001) in the liver, while at 3 mg/kg Cr-Pic it caused increased activity of this enzyme in breast muscle (*p* = 0.003). Lower CAT activity (*p* < 0.001) in chickens’ livers was noted in groups receiving 3 and 6 mg/kg Cr-NP and 3 mg/kg Cr-Pic, while higher activity in the group receiving 6 mg/kg Cr-Pic compared to the control group. Compared to the control group, the addition of Cr-Pic at 3 mg/kg caused a decrease in CAT activity (*p* = 0.038) in breast muscle, while at 6 mg/kg, it caused an increase in activity of this enzyme. In the liver of chickens receiving Cr at 3 mg/kg, SOD activity (*p* = 0.042) was higher than in the liver of chickens receiving Cr at 6 mg/kg. Increasing the addition of Cr from 3 to 6 mg/kg of feed caused an increase in CAT activity in liver and breast muscle (*p* < 0.001, both). In the liver of chickens from the Cr-NP treatment, higher SOD activity and lower CAT activity were observed than in the chickens from the Cr-Pic treatment (*p* < 0.001, both). In the breast muscle of chickens from the Cr-NP treatment, SOD activity was lower (*p* = 0.042), while CAT activity was higher (*p* = 0.023) (Table 4).

## 4. Discussions

Our earlier research found that Cr supplementations both as Cr-Pic and Cr_NP resulted in lower BW, ADFI, and livability, and reduced antioxidant enzyme activity. Our previous research suggested that the lowest dose of Cr, 3 mg/kg, was toxic for broiler chickens [19]. However, many authors have found a beneficial effect of using various forms of Cr in chickens’ diets on production results even when using higher doses of Cr than 3 mg/kg [25,26]. The authors of many studies report that the addition of Cr to the diet of poultry at dose 200–1200 µg/kg increases the activity of cellular insulin [8,27,28,29]. In our study, the addition of Cr to the diet of chickens, irrespective of its form, resulted in an increase in insulin levels and a decrease in glucagon levels in the blood plasma. Moreover, increasing the dose was found to increase these changes in hormone levels. The changes in the level of both hormones were probably a response to the increased blood glucose observed in chickens receiving Cr as a diet supplement. The results of our research on blood glucose content in chickens have been published in a paper by Ognik et al. [19]. According to Yildiz et al. [30], Cr is a cofactor for insulin activity. This element is part of the oligopeptide chromodulin, which, by binding to the active site of the insulin receptor tyrosine kinase, plays a role in the auto-amplification of signaling of this hormone. Insulin regulates carbohydrate, fat, and protein metabolism, stimulating amino acid uptake and protein synthesis, as well as glucose utilization in tissues [4].

The increased plasma serotonin levels noted in our study in chickens receiving Cr in the diet, in the form of both Cr-Pic and Cr-NP, were likely the result of increased insulin levels. Insulin supports transport of the serotonin precursor tryptophan across the blood–brain barrier, owing to its ability to increase the uptake of branched chain amino acids (BCAAs) by muscles. Chromium, in the form of picolinate, indirectly affects this process by reducing branched chain amino acids in the blood serum and thus increasing the ratio of tryptophan to BCAAs [8,31,32]. Franklin and Odontiadis [27] reported increased sensitivity of central serotonin 2A (5-HT_2A_) receptors in rats fed a diet supplemented with chromium picolinate at dose 100 mg/kg. Our research also showed an increase in plasma dopamine levels in chickens receiving a diet with Cr in the amount of 3 mg/kg. Due to the interaction of serotonin and dopamine through direct synaptic connections [33,34] and through physical heterocomplexes of 5-HT_2A_ and DA2 receptors [35,36], it is possible that chromium may also indirectly affect dopamine synthesis via the 5-HT system. Literature data indicate that the addition of Cr to the diet of rats at dose 100 mg/kg increased both serotonin and noradrenaline levels [27]. Our study found with an increase in plasma serotonin levels in chickens receiving Cr in their diet, but in contrast with the results reported by Franklin [27], noradrenaline levels decreased.

The blood glucose level is regulated not only by insulin but also by the histamine system [37,38]. Our study showed a decrease in the plasma histamine level of chickens receiving Cr at 3 mg/kg, irrespective of the form used. It is likely that Cr, by increasing the plasma insulin level, causes a decrease in the concentration of histidine, which is a precursor of histamine. In addition to the direct involvement of histamine receptors in the regulation of blood glucose, several studies have shown that the activation or deactivation of histamine receptors appears to have a modulating role in the regulation of the blood glucose level [38].

Our study showed an increase in the T4 level in the plasma of chickens receiving Cr in the amount of 6 mg/kg Cr-Pic. Sahin et al. [13] noted an increase in the concentration of T3 and T4 in heat-stressed chickens fed a diet with 0.4 mg/kg g Cr in the form of Cr picolinate. Taha et al. [39] reported that the addition of Cr to water (30 mg/L) for chickens did not cause an increase in serum T3 or T4. These results are consistent with those obtained by Al-Mashhadani et al. [40], who found that dietary supplementation with chromium yeast (0.5–2 mg/kg) had no significant effect on T3 and T4 levels. According to Sahin et al. [9], the addition of Cr to the diet of chickens (220–1200 µg/kg) reared under stress improves growth performance by regulating thyroid hormone metabolism (increased secretion of T3 and T4). 

In our study, the addition of Cr to the diet of chickens increased the concentration of Ca and Fe and decreased the concentration of Cu and Zn in their blood plasma. Similarly, Gubajdullina et al. [41] found that supplementation of a chicken’s diet with chromium (III) oxide nanoparticles at 50–400 µg/kg caused an increase in Fe and Ca content, while a decrease in Zn and Cu content in the body of birds. Iron is a cofactor for tyrosine hydroxylase and tryptophan hydroxylase, enzymes that are responsible for dopamine and serotonin synthesis, respectively [42], while magnesium affects norepinephrine level [43]. Uyanik et al. [44] found that the addition of CrCl_3_ to the diet of Japanese quail at 100 mg/kg did not affect the level of Ca, but increased the plasma Mg concentration. Similarly, Ebrahimzadeh et al. [15], after giving chickens chromium methionine chelate at 200, 400, and 800 ppb, noted no effect on the level of Ca and P in the blood plasma. Amatya et al. [45] found no effect of Cr supplementation in the form of potassium chromate, chromium chloride, or chromium-yeast complex on Zn and Fe levels in chicken blood plasma, but noted an increase in Cu levels. Research by Onderci et al. [16] indicates that the addition of Cr to the diet of laying hens increases the level of Fe in the blood, but also increases the level of Zn.

There is high individual variation among birds, which means that results of enzyme activity analyses fall within broad ranges. This poses significant difficulties in establishing physiological norms for enzyme activity in this group of animals. Their activity can increase both in the case of oxidative stress and under the influence of beneficial stimulation of the antioxidant system. To properly interpret the direction of changes, special attention should be paid to the level of other indicators, including MDA [46]. Our research shows that the addition of Cr to the diet of chickens at both 3 and 6 mg/kg, irrespective of the form used, increased MDA levels in the liver. The higher dose of Cr, i.e., 6 mg/kg, increased lipid peroxidation in both the liver and breast muscle, as evidenced by increased LOOH levels. The changes in the activity of antioxidant enzymes noted in our study (reduced SOD activity and higher CAT activity in the liver and breast muscle in the case of the higher dose of Cr) were the body’s response to the intensification of oxidative processes due to the addition of Cr to the diet. The increase in oxidative processes in both the liver and breast muscle was greater when Cr was used in the form of nanoparticles. According to Onderci et al. [16], Cr exhibits antioxidant capacity, which the authors demonstrated in a study on laying hens exposed to heat stress. The authors noted lower MDA levels in the blood of stressed laying hens receiving a Cr supplement. Our research has shown that the effect of a Cr supplement on redox status varied in different tissues: It was found to have an antioxidant effect in the blood [24], but a pro-oxidative effect in the liver and breast muscle. There are reports confirming the pro-oxidative effect of Cr. According to Fan et al. [47], the addition of Cr to the diet of chickens causes an increase in MDA levels and a decrease in antioxidant enzyme activity. The varied impact of Cr on redox status may depend on the length of exposure and the possibility of accumulation of this element in a given tissue. Changes noted in the activity of antioxidant enzymes may also be linked to the level of microelements, such as Fe, Cu, and Zn. Copper and zinc are SOD cofactors, while Fe is a CAT cofactor [46]. Reducing the level of micronutrients may be the result of increased use of these enzymes in antioxidant reactions.

## 5. Conclusions

The addition of Cr in the form of both Cr-Pic and Cr-NP was found to increase the level of serotonin at a dose of just 3 mg/kg and, at the same time, reduce the level of noradrenaline. The addition of Cr at 3 mg/kg, irrespective of the form used, regulated the level of hormones of carbohydrate metabolism (increasing insulin levels and reducing glucagon levels) and had an adverse effect on the antioxidant status of the liver and breast muscle. Due to the adverse effect of Cr at 3 mg/kg on the antioxidant status of chickens, this level of Cr should not be considered in both forms Cr-Pic and Cr-NP as a feed additive for broiler chickens. In the future, studies on the potential beneficial effects of Cr on the organism should take into account doses lower than 3 mg/kg.

## Figures and Tables

**Table 1:** Composition and nutrient density of the diets.

	Days 1–21	Days 22–35
Components, g/kg		
Maize	200.0	200.0
Soybean meal	336.5	282.4
Wheat	383.4	421.4
Soybean oil	39.0	56.1
Salt	3.3	3.3
Limestone	11.9	11.6
MCP	14.4	13.3
DL-Methionine	3.1	2.8
L-Lysine HCL	2.7	3.1
L-Threonine	0.7	1.0
Vitamins + trace minerals ^1^	5.0	5.0
Calculated nutrient density, g/kg		
Crude protein	220.0	200.0
Lysine	13.0	12.0
Methionine	6.2	5.7
Met. + Cys.	10.0	9.2
Threonine	8.5	8.0
Calcium	9.5	9.0
Available phosphorus	4.8	4.5
ME, kcal/kg	2950	3100
Amount of Cr added to feed	Analyzed content of Cr, mg/kg	
0	0.86	0.83
3 mg/kg Cr-Pic	3.90	3.36
6 mg/kg Cr-Pic	6.71	6.20
3 mg/kg Cr-NP	3.85	3.87
6 mg/kg Cr-NP	6.49	6.08

^1^ Provided per kilogram of diet: days 1 to 21: vit. A, 15,000 IU; vit. D_3_, 5000 IU; vit. E, 112 IU; vit. K_3_, 4 mg; vit. B_1_, 3 mg; vit. B_2_, 8 mg; vitamin B_6_, 5 mg; vit. B_12_, 16 mg; folic acid, 2 mg; biotin, 0.2 mg; nicotinic acid, 60 mg; calcium pantothenicum, 18 mg; choline, 1.8 g; Mn, 100 mg; Zn, 80 mg; Fe, 80 mg; Cu, 8 mg; I, 1 mg; Se, 0.15 mg; days 22 to 35: vit. A, 12,000 IU; vit. D_3_, 5000 IU; vit. E, 75 IU; vit. K_3_, 2 mg; vit. B_1_, 2 mg; vit. B_2_, 6 mg; vit. B_6_, 4 mg; vit. B_12_, 16 mg; folic acid, 1.75 mg; biotin, 0.05 mg; nicotinic acid, 60 mg; calcium pantothenicum, 18 mg; choline, 1.6 g; Mn, 100 mg; Zn, 80 mg; Fe, 80 mg; Cu, 8 mg; I, 1 mg; Se, 0.15 mg. ME – metabolic energy.

**Table 2:** Content of hormones in the blood plasma of chickens.

Treatment ^1,2^	Insulin ng/mL	Glucagon pg/mL	Serotonin ng/mL	Dopamine pg/mL	Noradrenaline ng/mL	Histamine ng/mL	T3 ng/mL	T4 ng/mL
Control	0.487	51.682	118.503	479.449	2.586	18.892	3.391	48.463
3 mg/kg Cr-Pic	0.568 ^b^	45.797 *	142.982 *^c^	479.682	2.233 *	14.967 *	3.676	52.562
6 mg/kg Cr-Pic	0.576 ^b^	45.303 *	147.089 *^c^	450.686	2.207 *	16.937*	3.769	53.952 *
3 mg/kg Cr-NP	0.581 ^b^	45.465 *	166.645 *^b^	538.004 *	2.180 *	11.517 *	3.332	53.260
6 mg/kg Cr-NP	0.945 *^a^	48.037	212.522 *^a^	537.471 *	2.195 *	19.842	3.461	53.596
SEM	0.033	0.723	5.067	7.019	0.039	0.517	0.078	0.844
Dose effect (D)								
3 mg/kg	0.575	45.631	154.813	508.843	2.207	13.242	3.504	52.911
6 mg/kg	0.761	46.670	179.806	494.078	2.201	18.389	3.615	53.774
Source effect (S)								
Cr-Pic	0.572	45.550	145.036	465.184	2.220	15.952	3.722	53.257
Cr-NP	0.763	46.751	189.583	537.737	2.187	15.679	3.397	53.428
*p*-value								
Control vs. all others	<0.001	0.016	<0.001	<0.001	0.001	<0.001	0.341	0.029
D effect	<0.001	0.421	<0.001	0.157	0.935	<0.001	0.534	0.637
S effect	<0.001	0.354	<0.001	<0.001	0.658	0.570	0.076	0.925
D × S interaction	0.001	0.238	<0.001	0.172	0.778	0.101	0.920	0.773

^1^ Diet supplemented with 6 and 3mg/kg Cr in the form of chromium picolinate (3 mg/kg Cr-Pic, 6 mg/kg Cr-Pic) or 6 and 3mg/kg Cr in the form of chromium nanoparticles (3 mg/kg Cr-NP, 6 mg/kg Cr-NP) and 0, without Cr supplementation. ^2^ Data represent mean values of nine replicate pens per treatment (1 chicken per pen). * Means within the same column differ significantly from the control at *p* ≤ 0.05. ^a–c^ Means within the same column differ significantly (*p* ≤ 0.05) according to Newman–Keuls mean comparison (only in the case of significant D × S interaction).

**Table 3:** Content of minerals in the blood plasma of chickens.

Treatment ^1,2^	Ca mmol/L	Mg mmol/L	Fe µmol/L	Cu µmol/L	Zn µmol/L
Control	1.662	0.827	6.140	6.252	24.136
3 mg/kg Cr-Pic	2.604 *	0.637 *	9.024 *	6.155	20.428 *
6 mg/kg Cr-Pic	2.676 *	0.589 *	10.445 *	4.803 *	20.752 *
3 mg/kg Cr-NP	2.852 *	0.680 *	10.588 *	5.351 *	19.359 *
6 mg/kg Cr-NP	2.298 *	0.653 *	11.668 *	4.388 *	19.930 *
SEM	0.092	0.020	0.307	0.129	0.281
Dose effect (D)					
3 mg/kg	2.728	0.658	9.806	5.753	19.894
6 mg/kg	2.487	0.621	11.057	4.595	20.342
Source effect (S)					
Cr-Pic	2.640	0.613	9.735	5.479	20.591
Cr-NP	2.575	0.667	11.128	4.869	19.645
*p*-value					
Control vs. all others	<0.001	0.001	<0.001	<0.001	<0.001
D effect	0.136	0.283	<0.001	<0.001	0.061
S effect	0.683	0.125	<0.001	<0.001	<0.001
D × S interaction	0.055	0.757	0.462	0.248	0.596

^1^ Diet supplemented with 6 and 3mg/kg Cr in the form of chromium picolinate (3 mg/kg Cr-Pic, 6 mg/kg Cr-Pic) or 6 and 3mg/kg Cr in the form of chromium nanoparticles (3 mg/kg Cr-NP, 6 mg/kg Cr-NP). ^2^ Data represent mean values of nine replicate pens per treatment (1 chicken per pen). * Means within the same column differ significantly from the control at *p* ≤ 0.05.

**Table 4:** Content of redox indicators in the liver and breast muscle of the chickens receiving different sources of Cr.

Treatment ^1,2^	LOOH, Μmol/Kg		MDA, Μmol/Kg		SOD, U/G Protein		CAT, U/G Protein	
	Liver	Breast Muscle	Liver	Breast Muscle	Liver	Breast Muscle	Liver	Breast Muscle
Control	6.243	2.194	0.805	0.906	1037.8	285.0	708.2	154.8
3 mg/kg Cr-Pic	4.859 *	2.062	0.781	0.825	920.6 *	308.7 *	621.6 *	136.5 *
6 mg/kg Cr-Pic	6.614	2.345 *	1.147*	0.963	871.6 *	256.6	954.4 *	185.5 *
3 mg/kg Cr-NP	6.153	2.337 *	1.256 *	1.121 *	1009.2	234.9	543.2 *	158.7
6 mg/kg Cr-NP	6.467	2.365 *	1.330 *	1.177 *	976.6	246.8	663.6 *	199.7 *
SEM	0.035	0.016	0.017	0.009	0.307	0.235	0.121	0.082
Dose effect (D)								
3 mg/kg	5.506	2.199	1.018	0.973	964.9	271.8	582.4	147.6
6 mg/kg	6.540	2.355	1.238	1.070	924.1	251.7	809.0	192.6
Source effect (S)								
Cr-Pic	5.736	2.203	0.964	0.894	896.1	282.6	788.0	161.0
Cr-NP	6.310	2.351	1.293	1.149	992.9	240.8	603.4	179.2
*p*-value								
Control vs. all	0.023	0.008	0.004	0.043	<0.001	0.003	<0.001	0.038
D effect	0.031	0.092	0.012	0.083	0.042	0.103	<0.001	<0.001
S effect	0.002	0.006	0.003	0.033	<0.001	0.042	<0.001	0.023
D × S interaction	0.233	0.342	0.865	0.435	0.338	0.132	0.114	0.226

^1^ Diet supplemented with 6 and 3 mg/kg Cr in the form of chromium picolinate (3 mg/kg Cr-Pic, 6 mg/kg Cr-Pic) or 6 and 3 mg/kg Cr in the form of chromium nanoparticles (3 mg/kg Cr-NP, 6 mg/kg Cr-NP). ^2^ Data represent mean values of nine replicate pens per treatment (1 chicken per pen) * Means within the same column differ significantly from the control at *p* ≤ 0.05. LOOH—lipid peroxides, MDA —malondialdehyde, SOD—superoxide dismutase, CAT—catalase.
